# Erythema Migrans in Patients with Post-Traumatic Splenectomy

**DOI:** 10.3390/microorganisms12071465

**Published:** 2024-07-19

**Authors:** Vera Maraspin, Katarina Ogrinc, Petra Bogovič, Tereza Rojko, Eva Ružić-Sabljić, Gary P. Wormser, Franc Strle

**Affiliations:** 1 Department of Infectious Diseases, University Medical Centre Ljubljana, 1525 Ljubljana, Slovenia; vera.maraspin@kclj.si (V.M.); katarina.ogrinc@kclj.si (K.O.); petra.bogovic@kclj.si (P.B.); tereza.rojko@kclj.si (T.R.); 2 Institute of Microbiology and Immunology, Medical Faculty, University of Ljubljana, 1000 Ljubljana, Slovenia; eva.ruzic-sabljic@mf.uni-lj.si; 3 Department of Medicine, Division of Infectious Diseases, New York Medical College, Valhalla, NY 10595, USA; gwormser@nymc.edu

**Keywords:** erythema migrans, Lyme borreliosis, Lyme disease, splenectomy, impaired immunity, treatment failure

## Abstract

Information on asplenic Lyme borreliosis (LB) patients with erythema migrans (EM) is lacking. We compared the course and outcome of 26 EM episodes in 24 post-trauma splenectomized patients (median age 51 years) diagnosed at a single clinical center in Slovenia during 1994–2023 with those of 52 age- and sex-matched patients with EM but with no history of splenectomy. All patients were followed for one year. A comparison of pre-treatment characteristics revealed that EM in splenectomized patients was of shorter duration before diagnosis (4 vs. 8 days, *p* = 0.034) with a smaller EM diameter (10.5 vs. 14 cm, *p* = 0.046), and more frequently fulfilled criteria for disseminated LB (3/26, 11.5% vs. 0%, *p* = 0.034). Treatment failure occurred in 5/26 (19.2%) EM episodes in splenectomized patients versus 0/52 in non-splenectomized patients (*p* = 0.003). The five treatment failure cases were retreated with antibiotic regimens used to treat EM and had complete resolution of all symptoms/signs. In conclusion, our study showed that splenectomized adult patients with EM differ somewhat in presentation and more often have treatment failure compared with non-splenectomized patients with EM.

## 1. Introduction

Lyme borreliosis (LB) is a zoonosis caused by spirochetes of the *Borrelia burgdorferi* sensu lato complex [1,2,3,4,5]. It is the most frequent tick-transmitted disease in the Northern Hemisphere [1,4,6,7,8,9]. LB usually begins with a skin lesion that develops at the site of a *Borrelia*-infected tick bite, days to a few weeks after the bite. Since the skin redness slowly expands outward, it is called erythema migrans (EM) [1,2,3,4,5]. The development of the EM skin lesion occurs as a result of cutaneous inflammation associated with the centrifugal spread of the spirochete from the bite site [4,10,11,12]. In untreated patients, *Borrelia* may spread (hematogenously) from the affected skin, leading to the development of secondary skin lesions (multiple EM) or to extracutaneous manifestations of LB. The propensity for dissemination depends on the characteristics of the *Borrelia* species causing the infection and on the (immune) ability of the host to resist the infection [2,3,4,5,13,14,15]. LB patients with spirochetemia are rarely febrile, unlike what is typically found with other bacteria that spread hematogenously in humans. The absence of lipopolysaccharide within the borrelial cell wall might be the explanation for this difference [4,16].

The host–pathogen interactions that occur in the skin probably have a critical role in determining the course and outcome of the infection [16,17]. The presence of T cells and the increased numbers of Langerhans cells at the site of the skin infection suggest that cell-mediated immune mechanisms are involved in the initial host response to *Borrelia*. High levels of mRNA expression of the T cell-active chemokines CXCL9 and CXCL10 and low levels of the B cell-active chemokine CXCL13 have been demonstrated in EM skin lesions, and CD3(+) T cells have been visualized using immunohistologic methods [16,18]. The global transcriptional alterations in skin biopsy samples of EM lesions from untreated adult US patients with Lyme disease in comparison to controls have revealed more than 250 differentially regulated genes characterized by the induction of chemokines, cytokines, Toll-like receptors, antimicrobial peptides, monocytoid cell activation markers, and numerous genes annotated as interferon (IFN)-inducible. The IFN-inducible genes included three transcripts involved in tryptophan catabolism (IDO1, KMO, KYNU) that play a pivotal role in immune evasion by certain other microbial pathogens [19].

Over the past decades, many clinical aspects of LB have been elucidated. However, data on the course of the disease in patients with impaired immunity are still scarce. In general, immunocompromised patients have an increased risk for the development of infection and for having a more severe illness, as well as for reactivation of the infection [20,21]. However, immunocompromised conditions are diverse, and the course of an infection depends on the type and degree of immune deficiency [20,21]. Post-traumatic splenectomy represents a special subgroup of altered immunity, which is mainly associated with complications in cases of intra-erythrocytic infections and infections with encapsulated bacteria.

Fifty-eight percent of Slovenia is covered by forests. Tick bites are common, and tick-transmitted diseases (such as LB and tick-borne encephalitis) are frequent. Slovenia is a highly endemic country for LB. In the period 2012–2021, the reported average incidence rate was 242 cases of LB per 100,000 inhabitants (maximal incidence >350/100,000 inhabitants per year). These high incidence rates [22], combined with a busy LB Outpatient Clinic that has been operating at our institution for more than 30 years, has allowed us to gain insight into rare situations, including an assessment of the course and outcome of LB in asplenic patients.

## 2. Materials and Methods

Information was obtained from our database of 14,823 episodes of EM in patients aged ≥15 years, diagnosed at the LB Outpatient Clinic of the University Medical Centre Ljubljana, Slovenia, in the years 1994–2023. For each patient, demographic, epidemiologic, and clinical data were collected using a structured questionnaire.

A solitary EM was defined as an expanding erythematous skin lesion, with or without central clearing, that developed days to weeks after a tick bite or exposure to ticks in an LB-endemic region and had a diameter of ≥5 cm. Multiple EM was defined as the presence of ≥2 skin lesions, at least 1 of which fulfilled the size criterion (≥5 cm) for a solitary EM [23]. Primary EM was defined as an EM that appeared at the site of a tick bite. If no tick bite was recalled, the primary EM was defined as the lesion with the longest duration, or in the case of the same or uncertain duration, the EM lesion with the largest diameter.

### 2.1. Selection of Patients and Control Group

Adult patients who presented with a typical EM skin lesion, had information about having had a previously removed spleen, and had at least a one-year follow-up qualified for the present study. However, since our primary aim was to assess the potential impact of splenectomy on the course and outcome of EM, we excluded patients who were immunocompromised due to other causes and in addition only included patients for whom the spleen was removed due to trauma and not because of underlying diseases.

The control group consisted of patients without any known immunodeficiency, who were diagnosed with EM in the same year that each of the splenectomized patients was diagnosed, and who were of the same sex, had the same or the most similar year of birth, received the same antibiotic therapy (the only exceptions to having received the same antibiotic regimen were for two episodes of EM in asplenic patients who were treated with ceftriaxone, whereas the matching non-splenectomized patients were instead treated with doxycycline) and were followed for one year; a 2:1 matching of controls to asplenic cases was performed. If more than two controls were identified fulfilling all required criteria, we chose the control whose name initials were the closest to the corresponding patient surname. The controls were not matched, however, based on the presence or absence of multiple EM skin lesions. Since multiple EM is the most common clinical manifestation of disseminated LB in Europe (much more common than Lyme neuroborreliosis, Lyme arthritis, and Lyme carditis) [5], such a matching would have made it impossible to assess differences in the proportion of disseminated LB between the two relatively small groups.

Over the 30 years of the current study, the assessment of disease course and outcome has remained virtually unchanged; however, any differences are explicitly noted below.

### 2.2. Re-Treatment Evaluation

#### 2.2.1. Clinical Evaluation

At the first visit, a comprehensive medical history was obtained and patients underwent a physical examination. Data on having had a tick bite, time from the bite to the onset of the skin lesion (as reported by the patient), time from the onset of the skin lesion to the diagnosis of EM at our LB Outpatient Clinic, the presence of concomitant local symptoms at the EM skin site (itching, burning, pain), and the presence of constitutional symptoms (fatigue, malaise, headache, myalgia, arthralgia, vertigo, fever) were assessed. Only symptoms that newly developed or worsened in comparison to pre-existing symptoms, that were temporally connected with the development of the EM skin lesion, and had no other known medical explanation qualified for being tabulated as EM-associated constitutional symptoms. The largest and the smallest diameters (in cm) of the EM skin lesion were measured at the first clinical evaluation before treatment with antibiotics and the area of skin involvement was calculated using the formula for the surface of the ellipse (longer diameter × shorter diameter × π/4) in square cm; furthermore, the appearance of the EM lesion was noted. All of this information was obtained using a questionnaire. Disseminated LB was defined based on the presence of ≥2 EM skin lesions or having an objective extracutaneous manifestation of LB.

Clinical re-evaluation was routinely performed at follow-up visits on day 14, and at 2–3, 6, and 12 months after the beginning of antibiotic treatment for EM. At the follow-up visit 14 days after the initiation of antibiotic therapy, patients were asked if they had been taking their medication regularly.

#### 2.2.2. Laboratory Analyses, Electrocardiogram, and Serologic Testing

Basic laboratory analyses (erythrocyte sedimentation rate, complete blood cell count, biochemical analyses) were performed at study entry and again 2 weeks later.

For all patients, an electrocardiogram was also performed at the first visit and repeated at the first follow-up visit on day 14 (or earlier if clinically indicated) if there were abnormal findings.

At each visit (with the exception of the follow-up visit at 2 weeks), testing for serum borrelial IgM and IgG antibodies was performed. From 1994 to 2011, an indirect immunofluorescent test (IFT) (positive result 1:256) was utilized; after 2011, an indirect chemiluminescence immunoassay (LIAISON, Diasorin, Saluggia, Italy) was performed instead with the results interpreted according to the manufacturer’s instructions.

#### 2.2.3. Cultivation and Identification of *Borrelia* Strains

Before antibiotic treatment, a classical skin biopsy (2.5 × 2 × 2 mm), or from 2010 onward a 3 mm punch skin biopsy, was performed on the border of EM skin lesion, and a whole-blood specimen (9 mL citrated blood) was obtained. Both the skin specimen and the blood sample were placed into Modified Kelly–Pettenkofer medium for up to 9 weeks in order to culture *Borrelia* spp. [24]. For patients with a positive skin culture, a second skin biopsy was performed 2–3 months later near the site of the original biopsy. Isolated *Borrelia* strains were characterized to the species level by MluI–large-restriction-fragment polymorphism (MluI-LRFP) [25,26].

### 2.3. Antibiotic Treatment

At the first visit, patients received oral or iv antibiotic regimens consistent with the guidelines at the time for the treatment of patients with EM, or instead based on the treatment trials the patients may have been enrolled in. EM episodes in the asplenic patients with a solitary EM were treated for 14 days with amoxicillin 500 mg tid (5 cases), cefuroxime axetil 500 mg bid (2 cases), or doxycycline 100 mg bid (5 cases), or for 5 days with azithromycin (1000 mg on day one, followed by 500 mg/day for 4 days; 9 cases). Asplenic patients with symptoms or signs suggestive of disseminated LB received a 14-day course of treatment with intravenous ceftriaxone 2 g once daily (3 cases) or with oral doxycycline 100 mg bid (2 cases). None of the patients were already receiving antibiotics at the time of the initial visit.

### 2.4. Assessment of the Course and Outcome

At the follow-up visits, the duration of the EM skin lesion after the initiation of antibiotic therapy was recorded, and the patients were asked about the presence and duration of other signs/symptoms that may have continued after the initiation of therapy. For this study, treatment failure was defined as the (i) appearance of an objective extracutaneous manifestation of LB following the completion of antibiotic therapy, (ii) the development or persistence of pronounced subjective symptoms that did not have an obvious alternative medical explanation, (iii) the persistence of EM at the examination conducted 2–3 months after the initiation of antibiotic treatment, or (iv) the demonstration of *Borrelia* by culture in a second skin biopsy specimen obtained at the site of the first biopsy at 2–3 months after initiation of treatment (rebiopsy was restricted to patients who initially had a positive skin biopsy culture). Patients with treatment failure were re-treated either with iv ceftriaxone or with a different oral antibiotic compared with the initial oral antibiotic treatment. Retreatment duration was 14 days.

### 2.5. Statistical Methods

Continuous variables were summarized using median values and ranges or interquartile ranges (IQRs), and discrete variables using counts and percentages with 95% confidence intervals. For discrete variables, all comparisons between groups were based on Fisher’s exact test. Differences in the duration of EM (in days) and the diameter of EM skin lesions were compared using the Wilcoxon rank-sum test. *p*-values < 0.05 were considered statistically significant.

All statistical analyses were performed using R software [27].

### 2.6. Ethics

This study was conducted in accordance with the Declaration of Helsinki, and was approved by the Medical Ethics Committee of the Republic of Slovenia (Project identification code 0120-78/2024-2711-3). The Ethics Committee waived the need for written informed consent.

## 3. Results

### 3.1. Patients with Splenectomy

**Figure 1:**
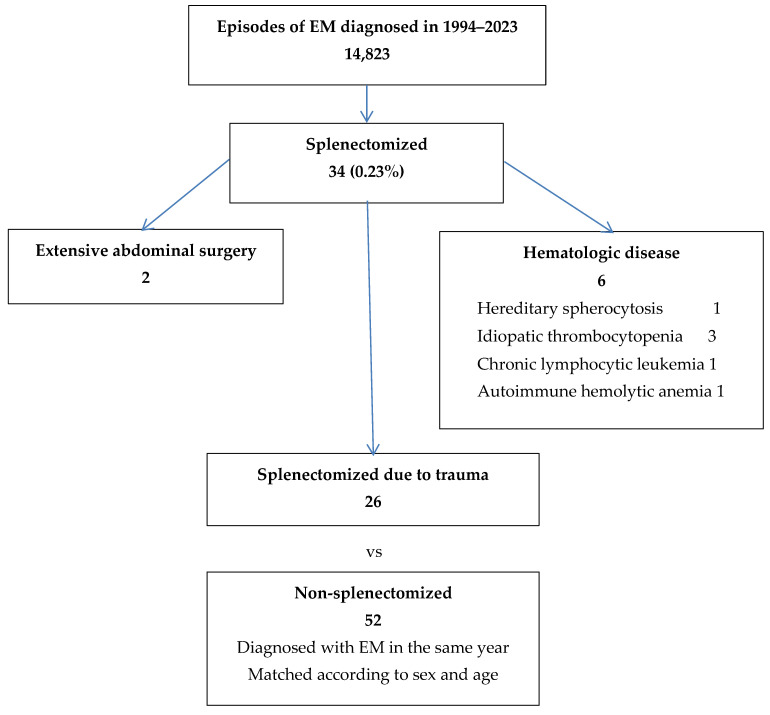
Description of the patient population with EM, with and without splenectomy.

In the 30-year period, 34/14,823 (0.23%) episodes of EM occurred in adult patients diagnosed at our institution who had had a prior splenectomy. For 26/34 (76.4%) cases, the splenectomy was trauma-related, in 6 (17.6%) patients it was performed because of an underlying hematologic disease (idiopathic thrombocytopenia—3 patients; chronic lymphocytic leukemia—1 patient; hereditary spherocytosis—1 patient; and autoimmune hemolytic anemia—1 patient), while in 2 (5.9%) patients the splenectomy was the result of extensive abdominal surgery (Figure 1).

In the present study, 26 episodes of EM in 24 patients with trauma-related splenectomy were enrolled. One of twenty-four patients had three episodes of EM several years apart from each other, whereas the others had only one episode of EM. The EM episodes occurred a median of 20 (range 1–55) years after the splenectomy. Five episodes occurred in females (19.2%) and 21 males (80.8%), with an overall median age of 51 (17–70) years at the time when the EM skin lesion was diagnosed. The basic demographic and clinical data for these 26 EM episodes in the 24 asplenic patients are summarized in Table 1.

One male patient with post-traumatic splenectomy performed in 1984 was diagnosed with and treated for a solitary EM three times at our LB Outpatient Clinic: in the years 1994, 2003, and 2005. In two of these three episodes, the patient was re-treated due to presumed treatment failure. In the 1994 EM episode, the patient was initially treated with iv ceftriaxone, 2 g per day, due to the presence of severe constitutional symptoms. The EM skin lesion, as well as the constitutional symptoms, disappeared during the 14-day course of antibiotic treatment. He was seronegative at presentation, with borderline IgM and IgG test results at the follow-up visit 2 months later. However, at 5 months after the onset of EM, a balance disorder with dizziness, vertigo, nausea, and vomiting began, and positive testing for borrelial IgG antibodies was detected. After re-treatment with another 14-day course of ceftriaxone, the symptoms completely resolved within 60 days. In 2003, the same patient presented with a 20 × 11 cm EM skin lesion that had developed on his left leg 17 days after a tick bite; mild constitutional symptoms were also present. He was seronegative at that time point, and the *Borrelia* skin culture was negative. He was treated with a 5-day course of azithromycin with a total dosage of 3 g over the 5 days. He was regarded as a treatment failure because the EM skin lesion was still visible at 56 days after the initial visit, prompting retreatment. After re-treatment with a 14-day course of doxycycline, the skin lesion completely disappeared within 16 days, with no residual symptoms. In addition, the course and outcome of the third EM episode diagnosed in 2005 was completely uneventful; for this EM episode, the patient had been treated with cefuroxime axetil.

### 3.2. Comparison of Splenectomized and Non-Splenectomized Patients

#### 3.2.1. Pre-Treatment Characteristics

Pre-treatment characteristics of EM in the splenectomized and non-splenectomized patients at the time of evaluation are shown in Table 2. Overall, 3/26 (11.5%) cases of early LB in splenectomized patients fulfilled the criteria for disseminated borrelial infection, in each case based on having multiple EM skin lesions.

The pre-treatment clinical characteristics of EM in asplenic patients in comparison to patients with spleens was similar for the majority of parameters assessed, including the proportion of patients recalling a tick bite at the site of the later EM, the incubation period (time from the bite to the onset of the EM skin lesion as reported by the patient), the characteristics of the EM skin lesion, the presence of associated symptoms, and the evaluated laboratory and microbiologic testing. However, episodes of EM in asplenic patients were of significantly shorter duration before diagnosis (4 vs. 8 days; *p* = 0.034) and had a significantly smaller lesion diameter (10.5 vs. 14 cm; *p* = 0.046). In addition, the asplenic patients more often reported headache (5/26, 19.2% vs. 2/52, 3.8%, *p* = 0.038), more often had an elevated (>10 × 10^9^/L) blood leukocyte count (5/26, 19.2% vs. 1/52, 1.9%; *p* = 0.014), and more frequently fulfilled the criteria for disseminated LB (3/26, 11.5% vs. 0%, *p* = 0.034).

#### 3.2.2. Post-Treatment Course and Outcome

There were no drop-outs in either the splenectomized or the control groups.

The duration of the EM skin lesion post-treatment was comparable in asplenic patients versus the control group of patients (median 11 vs. 11 days; *p* = 0.966).

In the majority of asplenic cases (21/26, 80.8%), including all 3 patients with early disseminated LB at enrollment (each of whom had multiple EM), the course and outcome of the illness were uneventful.

Treatment failure, however, occurred in 5/26 (19.2%) EM episodes in asplenic patients versus 0/52 in the control group (*p* = 0.003). Of the five treatment failures, two occurred in the same patient who was diagnosed with and treated for three separate EM episodes, as described above. Detailed information on the treatment failures is shown in Table 3. All patients with treatment failure were re-treated with either intravenous ceftriaxone (treatment duration = 14 days), or with a different oral antibiotic regimen (treatment duration = 14 days) compared with the oral antibiotic regimen that was used for the initial treatment, with a resolution of symptoms/signs and a favorable outcome of the illness within a 60-day time period after completion of retreatment (Table 3), and with follow-up extending to one year.

## 4. Discussion

For many centuries, the role of the spleen in human health has been poorly understood. In the early 20th century, a protective role of the spleen was suggested by a study in which there was higher mortality in splenectomized rats due to bacterial sepsis [28]. After 1952, when the problem of overwhelming post-splenectomy infections was described in a cohort of children with a previously removed spleen, it was recognized that asplenia may result in serious complications and even a fatal outcome [29]. Later on, the importance of the hematologic and immunologic functions of the spleen became appreciated [30]. Asplenia results in the impairment of phagocytic functions, dysfunction in both adaptive and innate immunity, and up-regulation of the inflammatory cascade [31]. Although infections with intra-erythrocytic parasites and encapsulated bacteria are the most frequent and well-known vulnerabilities for asplenic patients, the spleen probably also plays a role in the host’s immunologic response to other microorganisms [32,33,34,35]. Although over the past decades many aspects of LB have been clarified, information on the course and the outcome of early LB in asplenic patients is lacking. A PubMed database search for the articles published in English in the period 1990–2023, using the key words (“Lyme borreliosis” AND/OR “erythema migrans”) AND “splenectomy”, generated only two single case reports on babesiosis associated with Lyme disease co-infection [36,37]; however, in one of the reports, the diagnosis of Lyme disease was questionable. In addition, we found no descriptions of the disease course in asplenic patients with diseases caused by non-Lyme borrelia or by other spirochetes, such as *Treponema pallidum*, and only one description of leptospirosis in a splenectomized patient [38].

In the United States, splenectomy is performed in ~27,000 patients annually, which is approximately 8.2/100,000 inhabitants [39]. In Slovenia, a small country with 2.1 million inhabitants, in a 14-year period (from 2004 to 2017), 2167 patients were splenectomized, with a median number of 155 (range 114–183) patients annually [40] or 7.7/100,000 inhabitants. During the 30-year period of the present study, 14,823 patients were diagnosed with EM at our institution; however, only 34 (0.23%) were previously splenectomized. Herein, we present the findings on the course and outcome of early LB in asplenic patients who had had a prior splenectomy because of trauma.

A comparison of the assessed pre-treatment clinical and laboratory parameters between splenectomized and non-splenectomized patients (Table 2) showed several differences. For some of them, plausible explanations exist; for the others, they do not. The shorter duration of EM before diagnosis in splenectomized patients could be a reflection of greater awareness of the risk for severe course of infections and consequent earlier seeking of medical evaluation in asplenic patients. A reasonable explanation for the finding that, compared to patients with a preserved spleen, splenectomized patients more frequently had headache and a tendency for more frequent systemic symptoms, and more frequently presented with symptoms/signs of disseminated LB (in our group, multiple EM skin lesions) could have been because splenectomized patients have a higher incidence and/or a longer duration of spirochetemia. However, there is no current evidence for such an explanation. Namely, in the present study, all blood cultures were negative for *Borrelia* in both splenectomized patients (0/19) and those with a preserved spleen (0/47), which parallels well with the findings that *Borrelia* are rarely found in the blood of adult LB patients in Europe [41,42]. Leukocytosis is rare in patients with early LB [16]. The results of the present study are consistent with this conclusion, as mild leukocytosis was present in only 1/52 (1.9%) patients with a preserved spleen, but was significantly more common in splenectomized patients (5/26, 19.2%), *p* = 0.014. A comparison of the course of borrelial infection revealed a similar duration of EM skin lesions after the beginning of treatment with antibiotics in asplenic patients versus the control group, but a significantly higher rate of treatment failure in asplenic patients (*p* = 0.003), suggesting the negative impact of the immune deficiency associated with asplenia on the outcome of early LB. One possible explanation for the treatment failures could be that the patients were not compliant with taking antibiotics. This is unlikely, as the patients were routinely asked at the 14-day follow-up visit after the initiation of antibiotic therapy if they were taking their medication regularly, and the answer was always affirmative. In addition, the theoretical possibility of not taking medication in patients with a removed spleen (only this group had treatment failure) would be at most the same as in those with a preserved spleen (no treatment failure in this group) or even lower, as people are taught after spleen removal that they may have more severe infections. The possibility that an awareness of the importance of timely antibiotic treatment is better in patients with a removed spleen than in those without a splenectomy was suggested by the finding that asplenic patients presented significantly earlier after the EM skin lesion was noticed (4 vs. 8 days, *p* = 0.027).

Data from the literature indicate that patients with trauma-related splenectomy have a lower risk for the development of overwhelming post-splenectomy infections than asplenic patients with a hematologic disease [43] and that the development of overwhelming post-splenectomy infections is inversely related to the time since splenectomy, i.e., such infections are most frequent in the first 3 years after removal of the spleen [43,44]. In the current study, the number of splenectomized patients was too small to reliably evaluate the relationships between the course of LB and the time since splenectomy, and of note, only 3/26 asplenic patients had EM within 3 years of the splenectomy. Nevertheless, all splenectomized patients with EM treatment failure in the current study were re-treated with ceftriaxone or with an oral antibiotic that differed from the oral antibiotic treatment originally received (with a duration of treatment identical to what is typically used for initial treatment), and the outcome of LB based on a one-year observational period post retreatment was excellent. This favorable outcome is similar to EM treatment failure results in an Austrian report [45], as well as in our patients with various other forms of immune deficiencies [46,47,48]. These results can be compared because we used identical approaches to assess the course and outcome of EM in post-traumatic splenectomy patients to what was carried out in previous studies of EM patients with other forms of immunodeficiency, because the main source of information was the EM patient data file, and because all of these studies were performed by the same research team. The use of ceftriaxone for the treatment of EM is unusual from today’s point of view [3,4,5,9,16,49,50,51,52,53], but 30 years ago some patients with EM (especially those with multiple skin lesions and those with marked constitutional problems or suspected Lyme neuroborreliosis) were treated with ceftriaxone.

The advantages of the present study are the uniform approach to the management of patients with early LB, including the same approach to antibiotic treatment for both immunocompromised and non-immunocompromised patients, throughout the whole 30-year study period; the exact matching of the splenectomized and non-splenectomized patients with regard to age, sex, and the year of onset of EM; and no missed evaluations in either the splenectomized or the control group. In spite of the 3-decade-long duration of the present study, however, we were able to enroll only 34 episodes of EM in splenectomized patients, and just 26 in patients who were splenectomized because of trauma. This relatively small number of EM episodes in asplenic patients is the main limitation of the present study and limits the power to find statistically significant differences. Nevertheless, our study revealed that both the frequency of pre-treatment disseminated LB, as well as the frequency of treatment failure in splenectomized patients, significantly exceeded that in the matched control group of non-splenectomized patients. While the signs for disseminated LB were unequivocal (all three splenectomized patients had multiple EM), there might be some doubt in regard to the assessment of treatment failure for the patient who developed a balance disorder with dizziness, vertigo, nausea and vomiting that began 5 months after the treatment of EM. Although the absence of other known causes was consistent with a borrelial etiology, a diagnosis of Lyme neuroborreliosis was not made, as the cerebrospinal fluid examination revealed no pleocytosis, and testing for intrathecal synthesis of borrelial antibodies was not performed. However, even if this case was not considered a treatment failure, there would still have been significantly more treatment failures in the splenectomy group than in the spleen-preserved group (4/26 vs. 0/52, *p* = 0.011, instead of 5/26 vs. 0/52, *p* = 0.003). Furthermore, all of our patients were adults, so the findings may not be relevant to children. In addition, the findings are valid for Central Europe, where the vast majority of EM cases are caused by *Borrelia afzelii*; therefore, whether our findings would pertain to parts of Europe where a higher proportion of early LB is caused by *Borrelia garinii* is unclear. However, this concern may be more relevant for North America where *Borrelia burgdorferi* sensu stricto is by far the most common cause of LB.

## 5. Conclusions

The occurrence of early LB appears to be a rare event in patients with splenectomy: in the 30-year period, only 34/14,823 (0.23%) episodes of typical EM were diagnosed in adult asplenic patients at our institution, of whom 26 were in patients splenectomized due to trauma. In our study, EM in patients splenectomized because of trauma more often had symptoms/signs of dissemination, as well as treatment failure, than did non-asplenic patients. However, re-treatment with standard durations of antibiotics, as described above, resulted in a favorable outcome of the illness.

## Figures and Tables

**Table 1:** Basic demographic and clinical data on 26 erythema migrans episodes in 24 patients splenectomized due to trauma.

Demographic Data (*n* = 26)		
Age (years), median (range)	51 (17–70)	
Male sex, number (%; 95% CI)	21 (80.8%; 60.7–93.5%)	
Time since splenectomy (No. of cases, %) (*n* = 26)		
<3 years	3 (11.5%)	6 (23.1%)
3–10 years	3 (11.5%)	
10–20 years	10 (38.5%)	
20–30 years	5 (19.2%)	
>30 years	5 (19.2%)	

CI, confidence interval.

**Table 2:** Basic demographic, clinical, laboratory, and microbiologic data for patients with erythema migrans: comparison of findings in 26 episodes of erythema migrans in patients splenectomized because of trauma and 52 erythema migrans episodes in non-splenectomized patients without a known or suspected immune deficiency.

Pre-Treatment Clinical Characteristics			
	Splenectomized Patients *n* = 26 No. (%, 95% CI) or Median (Range; IQR)	Control Group Patients *n* = 52 No. (%, 95% CI) or Median (Range; IQR)	*p*-Value
Age in years	51 (17–70; 41–60)	51 (17–70; 42–60.5)	0.832
Male sex	21 (80.8%, 60.7–93.5%)	42 (80.8%, 67.5–90.4%)	1.000
Underlying chronic illness	11 (42.3%, 23.4–63.1%)	15 (28.8%; 17.1–43.1)	0.309
History of prior LB	2 (7.7%, 1.0–25.1%)	7 (13.5%, 5.6–25.8%)	0.710
Tick bite ^a^	19 (73.1%, 52.2–88.4%)	31 (59.6%, 45.1–73.0%)	0.359
Incubation (days) ^b^	16 (1–166; 10–29.5)	14 (1–90; 6.5–30)	0.528
Duration of EM to diagnosis (days) ^c^	4 (1–32; 3–7)	8 (1–65; 4–18)	**0.027**
Location of EM:			
extremities	19 (73.1%, 52.2–88.4%)	28 (53.8%, 39.5–67.8%)	0.142
trunk	7 (26.9%, 11.6–47.8%)	23 (44.2%, 30.5–58.7%)	0.144
neck	0 (0%, 0–13.2%)	1 (1.9%, 0.1–10.3%)	/
Largest diameter of EM (cm) ^d^	10.5 (4–27; 8–15)	14 (4–56; 9–19.5)	**0.046**
Area of EM lesion (cm^2^) at initial visit ^d^	47.1 (11.8–289.0; 35.3–132.0)	101.3 (9.4–1123.9; 43.6–155.9)	0.056
Appearance of EM: homogenous	18 (69.2%, 48.2–85.7%)	31 (59.6%, 45.1–73.0%)	0.464
Local symptoms:	16 (61.5%, 40.6–79.8%)	27 (51.9%, 37.6–66.0%)	0.475
itching	14 (53.8%, 33.4–73.4%)	24 (46.2%, 32.2–60.5%)	0.632
burning	4 (15.4%, 4.4–34.9%)	4 (7.7%, 2.1–18.5%)	0.430
pain	3 (11.5%, 2.5–33.2%)	1 (1.9%, 0.1–10.3%)	0.105
Constitutional symptoms:	12 (46.2%, 26.6–66.6%)	13 (25%, 14.0–39.0%)	0.074
fatigue	6 (23.1%, 9.0–43.7%)	7 (13.5%, 5.6–25.8%)	0.340
headache	5 (19.2%, 6.6–39.4%)	2 (3.8%, 0.5–13.2%)	**0.038**
arthralgia	5 (19.2%, 6.6–39.4%)	3 (5.8%, 1.2–16.0%)	0.109
myalgia	4 (15.4%, 4.4–34.9%	5 (9.6%, 3.2–21.0%)	0.471
dizziness	1 (3.8%, 0.1–19.6%)	3 (5.8%, 1.2–16.0%)	1.000
fever	2 (7.7%, 1.0–25.1%)	0 (0%; 0–6.9%)	0.108
Signs of disseminated early LB ^e^	3 (11.5%, 2.5–36.2%)	0 (0%, 0–6.9%)	**0.034**
Pre-treatment laboratory and microbiologic findings			
No laboratory abnormalities	13 (50%, 29.9–70.1%)	25 (48.1%, 34.0–62.4%)	1.000
Increased ESR (>20 mm)	3/21 (14.3%, 3.1–36.3%)	5/45 (11.1%, 3.7–24.1%)	0.702
WBC > 10 × 10^9^/L	5 (19.2%, 6.6–39.4%)	1 (1.9%, 0.1–10.3%)	**0.014**
WBC < 4 × 10^9^/L	0 (0%; 0–13.2%)	0 (0%; 0–6.9%)	-
Pts < 140 × 10^9^/L	0 (0%; 0–13.2%)	1 (1.9%, 0.1–10.3%)	-
Abnormal liver enzymes	6 (23.1%, 9.0–43.7%)	21 (40.4%, 27.0–54.9%)	0.206
Positive *Borrelia* IgM and/or IgG antibody	8 (30.8%, 14.3–51.8%)	20 (38.5%, 25.3–53.0%)	0.619
Isolation of *Borrelia* from skin	7/20 (35%, 15.4–59.2%)	21/47 (44.7%, 30.2–59.9%)	0.591
Isolation of *Borrelia* from blood	0/19 (0%, 0–17.7%)	0/47 (0%, 0–7.6%)	-
Post-treatment course and outcome			
Duration of EM (days) ^f^	11 (2–84; 7–18)	11 (2–60; 7–21)	0.966
Treatment failure	5 (19.2%, 6.6–39.4%)	0 (0%, 0–6.9%)	**0.003**
Time period for resolution of symptoms/signs after completion of re-treatment (days)	39 (16–60; 28–50)	Not applicable	

CI: confidence interval; EM: erythema migrans; IQR: interquartile range; LB: Lyme borreliosis; EM: erythema migrans; ESR: erythrocyte sedimentation rate (normal range 0–19 mm/h); WBC: white blood cell count (normal range: 4–10 × 10^9^/L); Pts: platelets (normal range: 140–340 × 10^9^/L). ^a^ At the site of the later EM skin lesion. ^b^ Data for patients who recalled tick bites at the site of the later skin lesion. ^c^ At time of enrollment. ^d^ In patients with multiple EM, the diameter (area) of the primary EM was utilized. ^e^ All 3 patients had multiple EM. ^f^ After the beginning of antibiotic treatment. Statistically significant differences are printed in bold.

**Table 3:** Erythema migrans treatment failures in patients with post-traumatic splenectomy.

Patient Number	1 *	2 *	3	4	5
Year of EM episode	1994	2003	2009	1994	2014
Sex	M	M	F	M	F
Age of patient at EM episode (years)	33	40	52	47	51
Number of EM skin lesions	1	1	1	1	1
Years after splenectomy	10	19	12	1	30
Diameters of EM (cm)	8 × 3	20 × 11	6 × 4	23 × 16	5 × 4
Constitutional symptoms	Severe ^a^	Mild ^b^	Severe ^c^	None	None
*Borrelia* antibodies at presentation IgM/IgG	Neg/neg ^d^ (IFT)	Neg/neg (IFT)	Neg/neg (IFT)	128/128 ^d^ (IFT)	Pos/neg; (chemiluminescence test, Liaison)
Skin culture	Positive (not typed)	Negative	*Borrelia afzelii* ^e^	Negative	Negative
Initial antibiotic treatment	Ceftriaxone	Azithromycin	Ceftriaxone	Amoxicillin	Azithromycin
Duration of EM after initiation of antibiotic in days	8	72	10	10	84
Description of treatment failure	Development of pronounced subjective symptoms (vestibular balance disorder with dizziness, vertigo, nausea, vomiting) 5 months after the initial visit ^f^; no obvious other medical explanation	Persistence of EM at 56 days after the initial visit ^e,f^	Demonstration of *Borrelia afzelii* ^e^ by culture of a repeat skin biopsy obtained at the site of first biopsy 70 days after the initial visit ^f^	Development of pronounced subjective symptoms (headache, arthralgia) 4.5 months after the initial visit ^f^; no obvious other medical explanation	Persistence of EM at 58 days after the initial visit ^f^
Antibiotic used for re-treatment (duration 14 days)	Ceftriaxone IV ^g^	Doxycycline orally	Doxycycline orally	Ceftriaxone IV ^g^	Cefuroxime-axetil orally
Days to resolution of symptoms/signs	60	16	NA ^h^	50	28
Remarks	CSF examination was within normal limits				

M: male; F: female; EM, erythema migrans; Neg: negative; Pos: positive; IFT, immunofluorescent test; NA, not applicable. * Two different episodes of EM in the same patient. ^a^ malaise, fever (38 °C), headache, myalgia; ^b^ malaise, headache; ^c^ fatigue, malaise, headache, vertigo, nausea; ^d^ later seroconversion (patient number 1) or elevation to 256/512 (patient number 4); ^e^ type Ba Mla1; ^f^ and institution of antibiotic treatment; ^g^ the use of ceftriaxone for the treatment of EM is unusual from today’s point of view, but 30 years ago some patients with multiple skin lesions and those with marked constitutional problems or suspected Lyme neuroborreliosis were treated with ceftriaxone. ^h^ A third skin biopsy 120 days after the first procedure (50 days after the second skin biopsy) was *Borrelia* culture-negative.

## Data Availability

The original contributions presented in the study are included in the article; further inquiries can be directed to the corresponding author.
